# Factors associated with alcohol consumption and prescribed drugs with addiction potential among older women and men – the Nord-Trøndelag health study (HUNT2 and HUNT3), Norway, a population-based longitudinal study

**DOI:** 10.1186/s12877-019-1114-2

**Published:** 2019-04-18

**Authors:** Kjerstin Tevik, Geir Selbæk, Knut Engedal, Arnfinn Seim, Steinar Krokstad, Anne-S Helvik

**Affiliations:** 1 Norwegian National Advisory Unit on Ageing and Health, Vestfold Hospital Trust, Tønsberg, Norway; 2 General Practice Research Unit, Department of Public Health and Nursing, Faculty of Medicine and Health Sciences, Norwegian University of Science and Technology (NTNU), Trondheim, Norway; 3 The Research Centre for Age-related Functional Decline and Disease, Innlandet Hospital Trust, Ottestad, Norway; 4 Faculty of Medicine, Institute of Health and Society, University of Oslo, Oslo, Norway; 5 Department of Geriatric Medicine, Oslo University Hospital, Oslo, Norway; 6 HUNT Research Centre, Department of Public Health and Nursing, Faculty of Medicine and Health Sciences, Norwegian University of Science and Technology, (NTNU), Levanger, Norway; 7 Psychiatric Department, Levanger Hospital, Nord-Trøndelag Hospital Trust, Levanger, Norway; 8 St. Olavs University Hospital, Sluppen, Trondheim, Norway

**Keywords:** Addictive drugs, Ageing, Alcohol, Elderly, Drinkers, Drinking, HUNT, Longitudinal study, Psychotropic drugs, Substance misuse

## Abstract

**Background:**

Little is known about factors associated with alcohol consumption and use of drugs with addiction potential in older adults. The aim of this study was to explore the association between socio-demographic variables, physical and mental health and the later (11 years) use of frequent drinking, prescribed drugs with addiction potential and the possible combination of frequent drinking and being prescribed drugs with addiction potential in older adults (≥ 65 years).

**Methods:**

In this longitudinal study, we used data from two surveys of the Nord-Trøndelag Health Study (HUNT2 1995–1997 and HUNT3 2006–2008), a population based study in Norway. We totally included 10,656 individuals (5683 women) aged 54 years and older when they participated in HUNT2. Frequent drinking was defined as drinking alcohol 4 days or more per week. Data on prescribed drugs with addiction potential were drawn from the Norwegian Prescription Database. Drugs with addiction potential were defined as at least one prescription of benzodiazepines, z-hypnotics or opioids during one year for a minimum of two consecutive years between 2005 and 2009.

**Results:**

The typical frequent drinker in HUNT3 was younger, more educated, lived in urban areas, and reported smoking and drinking frequently in HUNT2 compared to the non-frequent drinker in HUNT3. The typical user of prescribed drugs with addiction potential in HUNT3 was an older woman who smoked and was in poor health, suffered from anxiety, had been hospitalized in the last 5 years and used anxiety or sleep medication every week or more often in HUNT2. The typical individual in HUNT3 with the possible combination of frequent drinking and being prescribed drugs with addiction potential had more education, smoked, drank frequently and used anxiety or sleep medication in HUNT2.

**Conclusion:**

Individuals who were identified as frequent drinkers in HUNT2 were more likely to be frequent drinkers in HUNT3, and to have the possible combination of frequent drinking and being prescribed drugs with addiction potential in HUNT3. Health care professionals need to be aware of use of alcohol among older adults using drugs with addiction potential.

**Electronic supplementary material:**

The online version of this article (10.1186/s12877-019-1114-2) contains supplementary material, which is available to authorized users.

## Background

Older adults often consume less alcohol than their younger peers [1], but alcohol consumption, both in quantity and frequency, has increased in recent decades among older adults in Nordic [2–4] and European countries [5–7] and in the USA [8, 9]. This is of concern, as heavy alcohol consumption is harmful [10–14] and older adults are more susceptible to the side effects of alcohol because of age-related changes in metabolism and body composition [12, 15]. In the coming decades, the number of older adults will grow, increasing the likelihood that the number of older adults with problematic alcohol consumption will increase as well [16]. Knowing which factors are associated with frequent alcohol consumption in old age can help identify older adults that might be at higher risk of this problem.

A large number of studies have shown that alcohol consumption in older adults is associated with men [9, 17–22], younger age [9, 17, 20–22], current smoking [9, 17–21] and past smoking [17–19], higher socio-economic status (i.e. more education, income and social status) [7, 9, 17, 18, 21, 22], friends’ approval of drinking [23], living in an urban setting [17], better health status [9, 18–20, 22, 24] and less cognitive impairment [20]. Some studies have found having a co-habitant [17, 19, 22, 24] is associated with alcohol consumption in older adults, while other studies have found not having a co-habitant [9, 18, 25, 26] is associated with alcohol consumption among older adults. Moreover, there is conflicting evidence regarding an association between alcohol consumption and depression in older adults [9, 20, 27].

With a few exceptions [23, 26], studies of factors associated with alcohol consumption in older adults have a cross-sectional design [7, 9, 17–22, 24, 25, 27]. This makes it difficult to draw conclusions about causality related to the importance of socio-demographic, physical and mental health factors for alcohol consumption [28].

Drugs with addiction potential are used frequently among older adults in Western countries [28–31]. This is of concern because the use of drugs with addiction potential is associated with adverse health consequences [32–34]. In the present study, we use the term “drugs with addiction potential” to describe the use of benzodiazepines (hypnotics/sedative or anxiolytics), z-hypnotics or opioids, and the term “addictive psychotropic drugs” to describe benzodiazepines (hypnotics/sedatives or anxiolytics) and z-hypnotics.

The use of addictive psychotropic drugs in older adults has been associated with higher age [18, 19, 30, 35], women [7, 18, 19, 30, 35–39], smoking [19, 35], living alone [18], lower education and lower social status [7, 19], but the findings regarding gender and living alone is somewhat conflicting [35, 40]. Use of addictive psychotropic drugs in older adults has been related to psychiatric condition in general [35, 37] and to specific diseases such as depression [35, 39, 40], anxiety [39], dementia [40] and sleep disorders [35, 39, 40]. These drugs has also been associated with poorer health status [7, 18, 19, 35, 39], comorbidity [36, 37, 41]*,* higher levels of self-reported pain and stress [37] and more medical consultations [41]*.* In older adults, the use of opioids is more likely in older individuals [18], among women [18, 19], in those with worse health status [7, 18, 19], lower social status [7, 19] and those using multiple medications (polypharmacy) [18]. Several studies investigating factors associated with use of drugs with addiction potential have had a longitudinal design [7, 35–37, 39, 40], which allows us to draw conclusions with greater certainty regarding causality of factors associated with use of drugs with addiction potential.

There is concern about the combined use of alcohol and drugs with addiction potential in older adults [12, 29, 38]. Older adults are more susceptible to side effects from this combined use due to age-related changes in the absorption, distribution and metabolism of alcohol and drugs with addiction potential [12]. Individuals may experience effects from the interaction between alcohol and drugs with addiction potential even at light to moderate drinking levels (e.g. 1–2 drinks per occasion) [12]. The combined use of these drugs and alcohol may cause side effects such as increased sedation, memory problems, impaired coordination and breathing difficulties, which in turn can result in a higher risk of falls, injuries, accidents and even death [12, 18, 29, 38, 42].

Cross-sectional studies have identified older age [18], men [38], poorer health status [19], living in rural areas [18], living alone [18], higher social status [18], polypharmacy [18, 19] and poor social network [18] as contributing factors to the possible combination of frequent drinking and use of drugs with addiction potential among older adults.

Nevertheless, little is known about factors associated with frequent alcohol consumption and/or use of drugs with addiction potential in older adults in Norway [30, 43–46] or internationally [9, 16, 19, 40, 41]. A better understanding of these factors can help health personnel identify older adults who may be at higher risk for this use. The aim of this study is to explore the association between socio-demographic variables, physical and mental health and the later use (11 years) of frequent drinking, prescribed drugs with addiction potential and the possible combination of frequent drinking and use of these drugs in older men and women.

## Methods

### Study setting, data sources and participants

The Nord-Trøndelag Health Study (HUNT) [47] is based in Nord-Trøndelag County in mid-Norway [48, 49]. In 2006, the county had 128,649 residents, approximately 97% of whom were Caucasian [48, 49]. The population of Nord-Trøndelag County is fairly representative of Norway’s total population in terms of age and gender distribution, mortality and health status [48, 50]. The mean income and prevalence of more years of education in inhabitants, and the prevalence of current smokers are all slightly lower in Nord-Trøndelag compared to the whole of Norway [48]. The county also does not contain any large cities [48, 51].

Three HUNT Surveys have been completed to date: HUNT1 in 1984–1986, HUNT2 in 1995–1997 and HUNT3 in 2006–2008 [49]. All county residents 20 years or older have been invited to participate in the surveys [49].

We used data from HUNT2 and HUNT3 in this study. Individuals who were invited to participate in HUNT2 were also invited to participate in HUNT3 roughly 11 years later. Table 1 describes the total participation rates for HUNT2 and HUNT3, and for individuals aged 50 years and older. A non-participation study showed only minor potential non-participation bias after HUNT2, while non-participants in HUNT3 had lower socioeconomic status, poorer health, and a higher prevalence of chronic diseases and mental distress than those who participated [50]. Additional information on the HUNT studies has been provided elsewhere [48, 49].

**Table 1:** Participation in HUNT2 (1995–1997) and HUNT3 (2006–2008) Survey

HUNT2	Invited	Participated in HUNT2	
	N	N	%
Total^a^	93,898	65,237	69.5
Age^b^			
50-59	13,831^b^	11,205	81.2
60-69	10,655	9089	85.6
70–79	10,510	8310	79.9
80–89	5413	3202	66.0
90 +	906	387	52.9
HUNT3	Invited	Participated in HUNT3	
	N	N	%
Total^a^	93,860	50,807	54.1
Age^a^			
50-59	17,313	11,409	65.9
60–69	13,801	9811	71.1
70–79	8594	5744	66.8
80–89	5496	2287	41.6
90 +	890	153	17.2
Age at participation in HUNT2	Could have participated^c^ in HUNT3	Participated in both HUNT2 and HUNT3	
	N	N	%
Total^a^	65,237	37,071	56.8
Age^a^			
50-59	11,060	8195	74.1
60–69	9049	5049	55.8
70–79	7994	1800	22.5
80–89	2621	84	3.2
90 +	162	0	0

^a^Reference: Krokstad et al. [49]

^b^Reference: Holmen et al. [48]

^c^Could have participated: Includes those who participated in HUNT3, those who did not participate in HUNT3, those who were not invited (deceased, emigrated or disappeared), or moved out of the county

In our study, we included 10,656 participants aged 54 years and above from HUNT2 who had answered the first questionnaire in HUNT2 and the required question about alcohol consumption in HUNT3. Totally, 400 participants who responded to the alcohol frequency question in HUNT2, did not respond to this question in HUNT3, and thus excluded from the study. Very few participants who were 80 years or older in HUNT2 participated in HUNT3 (see Table 1). As they did not differ compared to those 79 years or younger in HUNT2 regarding frequent drinking or the possible combination of frequent drinking and use of drugs with addiction potential in HUNT3, they were included in our analyses. Due to missing information on individual independent variables in HUNT2, the number of participants varied from 10,656 to 6320 in our analyses.

Information about the use of drugs with addiction potential was drawn from the Norwegian Prescription Database (NorPD) [52] of the Norwegian Institute of Public Health from 2005 to 2009 and was linked to HUNT2 and HUNT3 participants. The NorPD contains data about dispensed drugs beginning in 2004 for all citizens in Norway [52].

### Measures

#### Alcohol consumption in HUNT2 and HUNT3

The drinking frequency measures in HUNT2 and HUNT3 differed slightly. In HUNT2, participants were asked how many times a month they drank alcohol, while participants in HUNT3 were asked how often during the last 12 months they drank alcohol. The response options in HUNT3 were: 1 = 4–7 times a week, 2 = 2–3 times a week, 3 = about once a week, 4 = 2–3 times a month, 5 = about once a month, 6 = a few times a year, 7 = not at all the last year and 8 = never drink alcohol. As drinking frequency was a categorical variable it was dichotomized in order to distinguish between those with high drinking frequency and those with lower drinking frequency or non-drinking. We defined drinking “4-7 times a week” as the equivalent of consuming alcohol 4 days or more per week in HUNT3 (alcohol consumption ≥ 4 days/week = frequent drinking/frequent drinkers). Our study considered drinking 16 times or more per month as the equivalent of consuming alcohol 4 days or more per week in HUNT2.

#### Drugs with addiction potential in HUNT2 and HUNT3

In HUNT2, participants self-reported use of prescribed drugs with addiction potential. They were asked to report how often they had taken anxiolytic or sleep medication in the last month, with the following response options: 1 = daily, 2 = weekly, but not every day, 3 = not as often as every week and 4 = never. Our study merged response categories 1 and 2 under the description of daily or weekly use of anxiolytic or sleep medication.

In the HUNT3 sample, information drawn from the NorPD (2005–2009) about prescribed drugs with addiction potential [52] was categorized according to the Anatomical Therapeutic Classification system (ATC) [53]. Drugs with addiction potential were defined as prescribed benzodiazepines (BZD), z-hypnotics or opioids. BZD were categorized under ATC codes N03AE (antiepileptic), N05BA (anxiolytic) and N05CD (hypnotic and sedative) [53]. Z-hypnotics were categorized under ATC code N05CF [53] and opioids under ATC code N02A [53]. In order to distinguish between use of drugs with addiction potential versus no use, the prescription of drugs with addiction potential was categorized as a dichotomized variable. The use of prescribed drugs with addiction potential was defined as at least one prescription of BZD, z-hypnotics or opioids within one year in a minimum of two consecutive years (2005/2006, 2006/2007, 2007/2008 or 2008/2009) [54, 55] which means that the drugs were prescribed at the same time as HUNT3 was completed.

To enable comparisons between prescribed drugs in HUNT2 and HUNT3, we defined a subgroup of addictive psychotropic drugs in HUNT3 as prescribed BZD or z-hypnotics.

#### Possible combination of use of alcohol and prescribed drugs with addiction potential in HUNT3

The possible combination of use of alcohol and prescribed drugs with addiction potential was defined as frequent drinking in HUNT3 and being prescribed drugs with addiction potential at the same time as HUNT3 was completed.

#### Independent variables from HUNT2

Smoking status in HUNT2 was assessed using three categories; 1 = never smoked daily, 2 = previously smoked daily and 3 = daily smoker.

Self-reported health status was assessed by one question with four response alternatives. Due to the response distribution, the variable was dichotomized to very good/good and poor/not so good health status.

Hospitalization during the last 5 years (yes/no) and history of medical diagnoses (myocardial infarction, angina, stroke/brain haemorrhage, diabetes, hyperthyroidism, hypothyroidism, osteoporosis, fibromyalgia, rheumatoid arthritis, epilepsy and asthma) were assessed by self-reported questions in HUNT2.

Anxiety and depression were self-reported by the Hospital Anxiety and Depression Scale (HADS) [56]. Seven items assess anxiety symptoms (HAD-A), and seven items assess depressive symptoms (HAD-D). Each item is scored 0–3, and the sum-score is from 0 to 21 on each subscale. A sum-score ≥ 8 on HAD-A and HAD-D indicates clinically significant anxiety (HAD-A) and depression (HAD-D), respectively [57]. The HADS has been validated in older adults [58] and in a general population [59] in Norway and the psychometric properties are good [58, 59].

Overall life satisfaction was assessed by one question with seven response categories ranging from 1 (very satisfied) to 7 (very dissatisfied). Due to the response distribution, the variable was dichotomized to “satisfied” (response option 1–3) and “dissatisfied/neither satisfied nor dissatisfied” (4–7).

Socio-demographic variables.

The socio-economic variables were gender, age at the time of survey completion, level of education (up to ten years of education, vocational and general education, college and university), urban versus rural living and marital status (living spouse or partner versus not). Age was further categorised into three groups (54–59 years, 60–64 years and 65 years or older).

#### Ethics and data protection

All participants signed an informed and written consent allowing use of their data for future medical research [48, 49] and allowing their data to be linked to other health records [48]. The linkage is possible due to the unique 11-digit personal identification number all Norwegians have [48]. In our study, the Norwegian Institute of Public Health linked data from HUNT2, HUNT3 and the NorPD, and only anonymous data were handed over for research due to Norwegian regulations.

The Regional Committee of Medical Research Ethics (REC) (reference number 2014/1248), the Norwegian Data Inspectorate Authority, the Norwegian Social Science Data Services (project number 40081), the Norwegian Data Inspectorate Authority (reference number 14/01248-2EOL) and the Norwegian Institute of Public Health have all approved the present study.

#### Statistics

The data was analysed with SPSS version 23. Descriptive analysis of categorical variables was performed using Chi-Square test.

We used McNemar’s Exact Test to investigate the change in drinking frequency (≥ 4 days/week) from HUNT2 to HUNT3 and the change in use of prescribed drugs; i.e. self-reported use of anxiolytic or sleep medication in HUNT2 to prescribed addictive psychotropic drugs in HUNT3.

Logistic regression analysis (the “enter” method) was used to examine the association between independent variables assessed in HUNT2 and three dependent variables assessed in HUNT3: Outcomes 1) frequent drinking (alcohol consumption ≥ 4 days/week) (versus not), 2) prescribed drugs with addiction potential (versus not), and 3) the possible combination of frequent drinking and being prescribed drugs with addiction potential (versus no frequent drinking, no use of drugs with addiction potential or neither frequent drinking nor being prescribed drugs with addiction potential). The independent variables (HUNT2) included in the unadjusted analyses were gender (women reference category), age (54–59 years reference category), level of education (up to ten years education reference category), living in urban versus rural areas, marital status (no living spouse or partner reference category), smoking status (never smoked daily reference category), overall health status (poor/not so good reference category), hospitalized during the last 5 years (versus not), HADS anxiety sum score ≥ 8 (versus < 8), HADS depression sum score ≥ 8 (versus < 8), overall life satisfaction (dissatisfied/neither satisfied nor dissatisfied reference category), frequent drinking (alcohol consumption ≥ 4 days/week) (versus alcohol consumption < 4 days/week) and self-reported use of anxiolytic and sleep medication every week or more (versus not). In the adjusted analyses, gender and age and other covariates associated with the outcome (*p* ≤ 0.1) in the unadjusted analyses were included.

The participants included in the assessment of outcome 3 (possible combined use of frequent drinking and use of drugs with addiction potential) were drawn from those included in outcome 1 (frequent drinking) and outcome 2 (drugs with addiction potential).

Statistical tests were carried out to assess for interactions between gender and other covariates. We did not find any interactions. Unadjusted and adjusted analyses were presented with odds ratios (OR) and 95% confidence intervals (CI). Probability values below 0.05 were considered statistically significant.

## Results

Table 2 shows the basic characteristics of HUNT2 participants (*N* = 10,656). The participants’ mean age was 62.7 (SD = 6.4) and 53% were women. The prevalence of self-reported medical illness was 4.0% for myocardial infarction, 6.3% for angina pectoris, 1.7% for stroke/brain haemorrhage, 2.5% for hyperthyroidism, 4.3% for hypothyroidism, 4.0% for osteoporosis, 5.0% for fibromyalgia, 3.6% for rheumatoid arthritis and 8% for asthma.

**Table 2:** Sample characteristics for the HUNT2 sample. The HUNT Study 1995–1997 (HUNT2) and 2006–2008 (HUNT3)

HUNT2		All participants	HUNT3	HUNT3	HUNT3
		Overall	Frequent drinking:alcohol consumption ≥ 4 days/week)^a^	Prescribed drugs with addiction potential^b^	Possible combination of frequent drinking (alcohol consumption ≥ 4 days/week) and being prescribed drugs with addiction potential^b^
Overall	N (%)	10,656 (100)	323 (3.0)	3444 (32.3)	107 (1.0)
Gender					
Women	N (%)	5683 (53.3)	114 (35.3)^d***^	2325 (67.5)^e***^	51 (47.7)^fNS^
Age					
53–59 years	N (%)	4318 (40.5)	134 (41.5)^d**^	1096 (31.8)^e***^	48 (44.9)^fNS^
60–64 years	N (%)	2677 (25.1)	93 (28.8)	838 (24.3)	21 (19.6)
65 +	N (%)	3661 (34.4)	96 (29.7)	1510 (43.8)	38 (35.5)
Level of education^c^					
Up to ten years education	N (%)	8421 (83.1)	166 (52.4)^d***^	2800 (86.3)^e***^	61 (59.2)^f***^
Vocational and general education	N (%)	255 (2.5)	23 (7.2)	73 (2.3)	6 (5.8)
College and university	N (%)	1454 (14.4)	128 (40.4)	371 (11.4)	36 (35.0)
Living in					
Rural areas	N (%)	4141 (38.9)	79 (24.5) ^d***^	1414 (41.1)^e***^	31 (29.0)^fNS^
Urban areas	N (%)	6515 (61.1)	244 (75.5)	2030 (58.9)	76 (71.0)
Marital status^c^					
No living spouse or partner	N (%)	2223 (20.9)	53 (16.4)^d*^	828 (24.1)^e***^	26 (24.3)^fNS^
Living spouse or partner	N (%)	8420 (79.1)	270 (83.6)	2611 (75.9)	81 (75.7)
Smoking status^c^					
Never smoked daily	N (%)	4607 (43.6)	81 (25.2)^d***^	1425 (41.9)^e***^	26 (24.5)^f***^
Former daily smoker	N (%)	3772 (35.7)	162 (50.3)	1141 (33.6)	50 (47.2)
Daily smoker	N (%)	2177 (20.7)	79 (24.5)	832 (24.5)	30 (28.3)
Overall health status^c^					
Poor/not quite good	N (%)	3624 (34.3)	84 (26.2)^d**^	1580 (46.2)^e***^	43 (40.2)^fNS^
Good/very good	N (%)	6940 (65.7)	237 (73.8)	1838 (53.8)	64 (59.8)
Hospitalized during the last 5 years^c^	N (%)	3051 (33.2)	92 (32.4)^dNS^	1144 (38.5)^e***^	36 (38.3)^fNS^
HADS anxiety score ≥ 8^c^	N (%)	1091 (12.9)	30 (10.8)^dNS^	542 (20.8)^e***^	15 (17.4)^fNS^
HADS depression score ≥ 8^c^	N (%)	1166 (12.1)	29 (9.6)^dNS^	494 (16.0)^e***^	13 (13.3)^fNS^
Life satisfaction^c^					
Dissatisfied/neither satisfied nor dissatisfied	N (%)	1586 (15.1)	39 (12.3)^dNS^	668 (19.7)^e***^	15 (14.4)^fNS^
Satisfied	N (%)	8904 (84.9)	277 (87.7)	2716 (80.3)	89 (85.6)

*HADS* Hospital Anxiety Depression scale, *NS* non-significant

^a^Self-reported alcohol consumption assessed among participants in HUNT3

^b^Information about prescribed drugs with addiction potential among participants in HUNT3 (2006–2008) was drawn from the Norwegian Prescription Database. Drugs with addiction potential were defined as at least one prescription of benzodiazepines, z-hypnotics or opioids in two consecutive years (2005/2006, 2006/2007, 2007/2008 or 2008/2009). Benzodiazepines defined by N03AE, N05BA and N05CD. Z-hypnotics defined by N05CF. Opioids defined by N02A

^c^Number do not sum up to 10,656 because of missing information

^d^Significance testing with Chi-square test between no frequent drinking (alcohol consumption < 4 days/week) and frequent drinking (alcohol consumption ≥4 days/week) (HUNT3)

^e^Significance testing with Chi-square test between no prescribed drugs with addiction potential and prescribed drugs with addiction potential (HUNT3)

^f^Significance testing with Chi-square test between “no possible combination of frequent drinking (alcohol consumption ≥ 4 days/week) and being prescribed drugs with addiction potential” and “possible combination of frequent drinking (alcohol consumption ≥ 4 days/week) and being prescribed drugs with addiction potential” (HUNT3)

*P < 0.05

**P < 0.01

***P < 0.001

### Frequent drinking in HUNT3

In total, 3.0% (323/10,656) of older adults (≥ 54 years in HUNT2) were frequent drinkers (alcohol consumption ≥ 4 days/week) when they participated in HUNT3 (Table 2). There was no association between any medical diagnosis in HUNT2 and frequent drinking in HUNT3, with the exception of rheumatoid arthritis. Participants in HUNT3 who were frequent drinkers (versus not), were less likely in HUNT2 to report rheumatoid arthritis (1.3% versus 3.7%).

In total, 4.8% (400/8355) of the participants who responded to the alcohol frequency question in HUNT2, were excluded in HUNT3 since they did not respond to the alcohol frequency question in HUNT3 (Additional file 1: Table S1). Those excluded did not differ in frequency of drinking in HUNT2, but were older, more often women, lived without a spouse or partner, were non-smokers, had poor health status, had more anxiety and used anxiolytic or sleep medication in HUNT2 and had fewer years of education compared to those who responded to the alcohol frequency question in HUNT3.

Among participants who had answered the alcohol frequency question in HUNT2 and HUNT3, the prevalence of frequent drinking increased in the total sample from 1.0% in HUNT2 to 3.7% in HUNT3. Among women, this increase was from 0.4 to 2.7% while among men it was from 1.7 to 4.7% (Table 3).

**Table 3:** Use of alcohol and prescribed drugs in HUNT2 (1995–1997) and HUNT3 (2006–2008)

		Prevalence of frequent drinking (alcohol consumption ≥ 4 days/week) among participants in HUNT2 and who participated in HUNT3 11 years later	
		HUNT2	HUNT3
Total (*n* = 7955)	N (%)	83 (1.0)	297 (3.7)^c***^
Gender			
Women (*n* = 3876)	N (%)	14 (0.4)	106 (2.7)^c***^
Men (*n* = 4079)	N (%)	69 (1.7)	191 (4.7)^c***^
Age group in HUNT2:			
53–59 years (*n* = 3462)	N (%)	30 (0.9)	150 (4.3)^c***^
60–64 years (*n* = 2020)	N (%)	20 (1.0)	71 (3.5)^c**^
≥ 65 years (*n* = 2473)	N (%)	33 (1.3)	76 (3.1)^c**^
		Prevalence of use of prescribed drugs among participants in HUNT2^a^ and who participated in HUNT3 11 years later^b^	
		HUNT2	HUNT3
Total (*n* = 8691)	N (%)	858 (9.9)	2354 (27.1)^c***^
Gender			
Female (*n* = 4714)	N (%)	621 (13.2)	1681 (35.7)^c***^
Male (*n* = 3977)	N (%)	237 (6.0)	673 (16.9)^c***^
Age group in HUNT2			
53–59 years (*n* = 3481)	N (%)	265 (7.6)	693 (19.9)^c***^
60–64 years (*n* = 2196)	N (%)	207 (9.4)	573 (26.1)^c***^
≥ 65 years (*n* = 3014)	N (%)	386 (12.8)	1088 (36.1)^c***^

^a^Information about prescribed drugs in HUNT2: Self-reported use of anxiolytic or sleep medication every week or more in the last month

^b^Information about prescribed drugs in HUNT3: Addictive psychotropic drugs drawn from the Norwegian Prescription Database were defined as at least one prescription of benzodiazepines or z-hypnotics in two consecutive years (2005/2006, 2006/2007, 2007/2008 or 2008/2009). Benzodiazepines defined by N03AE, N05BA and N05CD. Z-hypnotics defined by N05CF

^c^Significance testing with McNemar’s Exact Test

^***^P < 0.001

Frequent drinkers in HUNT2 had about 33 times higher odds of being frequent drinkers in HUNT3 compared to non-frequent drinkers in HUNT2 (Table 4).

**Table 4:** Adjusted logistic regression analyses of alcohol consumption and use of drugs with addiction potential (HUNT3)

HUNT2	Outcome 1 (HUNT3)^a^:	Outcome 2 (HUNT3)^a^:	Outcome 3 (HUNT3)^a^:
	Frequent drinking:	Prescribed drugs with	Possible combination of frequent drinking
	alcohol consumption ≥ 4 days/week^b^	addiction potential^c^	(alcohol consumption ≥4 days/week) and being prescribed drugs with addiction potential
	Adjusted^e^	Adjusted^e^	Adjusted^e^
	OR 95% CI	OR 95% CI	OR 95% CI
Gender (men)	1.22 (0.93–1.60)	**0.45 (0.40–0.51)**	0.77 (0.48–1.25)
Age			
54–59	1 (ref. category)	1 (ref. category)	1 (ref. category)
60–64	0.82 (0.60–1.12)	**1.43 (1.23–1.66)**	0.66 (0.35–1.27)
≥ 65	**0.69 (0.51–0.95)**	**2.06 (1.79–2.37)**	1.23 (0.74–2.05)
Level of education			
Up to ten years education	1 (ref. category)	1 (ref. category)	1 (ref. category)
Vocational and general education	**2.84 (1.63–4.95)**	0.89 (0.62–1.27)	1.23 (0.36–4.19)
College and university	**3.69 (2.81–4.86)**	0.90 (0.76–1.07)	**2.35 (1.40–3.95)**
Living in			
Rural areas	1 (ref. category)	1 (ref. category)	1 (ref. category)
Urban areas	**1.68 (1.26–2.25)**	0.92 (0.82–1.04)	1.33 (0.80–2.21)
Marital status			
No living spouse or partner	1 (ref. category)	1 (ref. category)	
Living spouse or partner	1.05 (0.74–1.48)	0.96 (0.83–1.11)	
Smoking status			
Never smoked daily	1 (ref. category)	1 (ref. category)	1 (ref. category)
Former daily smoker	**1.95 (1.43–2.65)**	**1.28 (1.11–1.46)**	**2.25 (1.25–4.06)**
Daily smoker	**1.49 (1.04–2.16)**	**1.69 (1.44–1.98)**	**1.98 (1.01–3.88)**
Overall health status			
Poor/not quite good	1 (ref. category)	1 (ref. category)	
Good/very good	1.07 (0.80–1.42**)**	**0.64 (0.56–0.73)**	
Hospitalized during the last 5 years		**1.21 (1.07–1.37)**	
HADS anxiety score			
< 8		1 (ref. category)	
≥ 8		**1.68 (1.39–2.03)**	
HADS depression score			
< 8		1 (ref. category)	
≥ 8		1.00 (0.81–1.23)	
Overall life satisfaction			
Dissatisfied/neither satisfied nor dissatisfied		1 (ref. category)	
Satisfied		1.02 (0.84–1.23)	
Alcohol consumption ^d^			
< 4 days/week	1 (ref. category)		1 (ref. category)
≥ 4 days/week	**33.32 (20.09–55.25)**		**8.40 (3.67–19.25)**
Use of anxiolytic or sleep medication every week or more^d^			
No		1 (ref. category)	1 (ref. category)
Yes		**5.67 (4.56–7.05)**	**2.25 (1.23–4.12)**
R^2^%^f^	17.3	19.5	6.8

Bold numbers indicate significant associations. *OR* odds ratio, *CI* confidence interval, *HADS* Hospital Anxiety and Depression scale

^a^Number of participants in the adjusted analyses with outcome 1 is *N* = 7541, outcome 2 *N* = 6277 and outcome 3 *N* = 6320

^b^Self-reported alcohol consumption assessed among participants in HUNT3 (2006–2008)

^c^Information on prescribed drugs with addiction potential among participants in HUNT3 (2006–2008) was drawn from the Norwegian Prescription Database. Drugs with addiction potential were defined as at least one prescription of benzodiazepines (BZD), z-hypnotics or opioids in two consecutive years (2005/2006, 2006/2007, 2007/2008 or 2008/2009). BZD defined by N03AE, N05BA and N05CD, z-hypnotics defined by N05CF and opioids by N02A

^d^Self-reported use of alcohol in the last month and self-reported use of anxiolytic or sleep medication every week or more in the last month among participants in HUNT2

^e^Adjusted binary logistic regression analysis. Gender and age and other covariates associated with the outcome (*p* ≤ 0.1) in the unadjusted analyses (see Supplemental Table 2) were included in the adjusted analyses. Outcome 1: frequent drinking (alcohol consumption ≥ 4 days/week) (alcohol consumption < 4 days/week reference category). Outcome 2: prescribed drugs with addiction potential (BZD, z-hypnotics or opioids) (no prescribed drugs with addiction potential reference category). Outcome 3: possible combination of frequent drinking (alcohol consumption ≥ 4 days/week) and being prescribed drugs with addiction potential (BZD, z-hypnotics or opioids). Reference category: no frequent drinking, no use of drugs with addiction potential or neither frequent drinking - nor being prescribed drugs with addiction potential

^f^Nagelkerke R-squared for the adjusted analyses

In adjusted logistic regression analyses, the typical frequent drinker in HUNT3 was younger, had more education, lived in urban areas, and smoked and drank frequently (≥ 4 days/week) in HUNT2 compared to the non-frequent drinker in HUNT3 (Table 4). The explained adjusted variance assessed using Nagelkerke R-Squared was 17.3%. Unadjusted results are shown in Additional file 2: Table S2.

### Drugs with addiction potential in HUNT3

Nearly one-third (3444/10,656 = 32.3%) of participants in HUNT3 were prescribed at least one drug with addiction potential (BZD, z-hypnotics or opioids) in two consecutive years (Table 2). About 10% reported use of anxiolytic or sleep medication in HUNT2, and 27.1% were prescribed addictive psychotropic drugs (BZD or z-hypnotics) in HUNT3 (Table 3).

Participants in HUNT3 who were prescribed drugs with addiction potential (versus not), were more likely in HUNT2 to report angina pectoris (8.0% versus 5.5%), stroke/brain haemorrhage (2.7% versus 1.2%), hyperthyroidism (3.2% versus 2.2%), hypothyroidism (5.7% versus 3.7%), osteoporosis (6.5% versus 2.9%), fibromyalgia (9.4% versus 3.1%), rheumatoid arthritis (5.3% versus 2.9%) and asthma (9.7% versus 7.2%).

Users of anxiolytic or sleep medication in HUNT2 had more than five times higher odds of being prescribed drugs with addiction potential in HUNT3 compared to non-users of anxiolytic or sleep medication in HUNT2 (Table 4).

In adjusted logistic regression analyses, the typical user of prescribed drugs with addiction potential in HUNT3 was found to be an older woman who smoked and was in poor health, suffered from anxiety, had been hospitalized during the last 5 years and had reported use of anxiety or sleep medication every week or more in HUNT2 (Table 4). The explained adjusted variance assessed using Nagelkerke R-squared was 19.5%. Unadjusted findings are presented in Additional file 2: Table S2.

### Possible combination of frequent drinking and being prescribed drugs with addiction potential in HUNT3

One per cent of the HUNT3 study sample (107/10,656) were frequent drinkers and had also been prescribed drugs with addiction potential during the same period that HUNT3 was completed (Table 2). With one exception, we found no association between any medical diagnosis in HUNT2 and the possible combination of frequent drinking and being prescribed drugs with addiction potential in HUNT3. The exception was that participants in HUNT3 with this possible combination (versus not), were more likely in HUNT2 to report angina pectoris (13.2% versus 6.2%).

Frequent drinkers in HUNT2 had about eight times higher odds of having the possible combination of frequent drinking and being prescribed drugs with addiction potential in HUNT3 compared to non-frequent drinkers in HUNT2 (Table 4). Users of anxiolytic or sleep medication in HUNT2 had about two times higher odds of having the possible combination of frequent drinking and being prescribed drugs with addiction potential in HUNT3 compared to non-users of anxiolytic or sleep medication in HUNT2 (Table 4).

In the adjusted logistic regression analyses, the typical individual with the possible combination of frequent drinking and being prescribed drugs with addiction potential in HUNT3 had more education, smoked, drank frequently and used anxiety or sleep medication in HUNT2 compared to the individual with no such use in HUNT3 (Table 4). The explained adjusted variance assessed using Nagelkerke R-squared was 6.8%. Unadjusted results are shown in Additional file 2: Table S2.

## Discussion

This is the first large population-based study to explore factors associated with frequent drinking (alcohol consumption ≥ 4 days/week) and being prescribed drugs with addiction potential (BZD, z-hypnotics or opioids) using a longitudinal design in older adults aged 65 years or more at follow-up.

In our study, the typical frequent drinker in HUNT3 was younger, had more years of education, lived in urban areas, and smoked and drank frequently (≥ 4 days/week) in HUNT2 compared to the non-frequent drinker in HUNT3. The typical user of prescribed drugs with addiction potential in HUNT3 was found to be an older woman who smoked and was in poor health and suffered from anxiety, had been hospitalized in the last 5 years and used anxiety or sleep medication every week or more in HUNT2. The typical individual with the possible combination of frequent drinking and being prescribed drugs with addiction potential in HUNT3 had more education, smoked, drank frequently and used anxiety or sleep medication in HUNT2 compared to the individual with no such use in HUNT3 (see Table 5 which summarize the findings).

**Table 5:** Factors associated with frequent drinking and drugs with addiction potential^a^. (HUNT2 1995–1997 and HUNT3 2006–2008)

Outcome 1 (HUNT3): Frequent drinking (≥ 4 days/week)	Outcome 2 (HUNT3): Use of prescribed drugs with addiction potential	Outcome 3 (HUNT3): Possible combination of frequent drinking (≥ 4 days/week) and use of prescribed drugs with addiction potential
Factors assessed in HUNT2:	Factors assessed in HUNT2:	Factors assessed in HUNT2:
• Younger age	• Women	• More years of education
• More years of education	• Older age	• Daily smoker or former daily smoker
• Living in urban areas	• Daily smoker or former daily smoker	
• Daily smoker or former daily smoker	• Poor health • Anxiety	• Frequent drinking (≥ 4 days/week)
• Frequent drinking (≥ 4 days/week)	• Been hospitalized in the last 5 years • Use of anxiolytic or sleep medication every week or more	• Use of anxiolytic or sleep medication every week or more

^a^Detailed information from adjusted analyses is found in Table 4

In our study, we found that those who were frequent drinkers (≥ 4 days/week) in HUNT2 had about 33 times higher odds of being frequent drinkers when they participated in HUNT3. Furthermore, they had about eight times higher odds of having the possible combination of frequent drinking and being prescribed drugs with addiction potential in HUNT3. Thus, it seems that middle-aged drinkers continue their drinking pattern of frequent drinking as they age.

Since our definition of alcohol drinking only included frequency of alcohol consumption (≥ 4 days/week), this definition may include participants with both risky drinking (≥ 7 drinks in a week) and non-risky drinking (< 7 drinks in a week) [60]. Thus, it is uncertain if higher odds for later frequent drinking will increase the risk of alcohol-related problems in older adults [60]. However, a recently published meta-analysis, including all ages, found that the level of alcohol consumption that minimizes health loss is zero [14]. Moreover, a combination of frequent drinking and use of drugs with addiction potential is of concern as greater drinking frequency increases the risk of concomitant use of alcohol and drugs [31, 61]. However, we do not know if these substances were used together in our study.

Though the majority of our sample were non-frequent drinkers (< 4 days/week), our study has shown that drinking frequency ≥ 4 days/week increased overall from HUNT2 to HUNT3, and in both genders. The increased number of healthy years experienced by older adults [6, 7], improved household finances [4], international travelling [4] and a more “continental” drinking pattern [43] might be reasons for the increase in drinking frequency among older adults in Norway. Even though the drinking pattern in HUNT2 of those not responding to the alcohol frequency question in HUNT3 (4.8%) did not differ with those responding to this question in HUNT3, the increased prevalence of high drinking frequency in HUNT3 might be inflated by those excluded. Those excluded were older, more often women, had fewer years of education and poorer health status and may have stopped drinking. We do not know why they did not answer the question. It could be because it was not relevant to them, albeit the alcohol frequency question did allow for a non-drinking response.

Furthermore, we found that the reported use of anxiolytic and sleep medication in HUNT2 was associated with being prescribed drugs with addiction potential, and the possible combination of frequent drinking and being prescribed drugs with addiction potential in HUNT3. Other studies have found similar results, where previous use of addictive psychotropic drugs was highly associated with subsequent use [37, 39]. Unfortunately, we do not know the indications for the prescriptions, the dosages or use between these two time points. Moreover, we found an increase in the use of addictive psychotropic drugs from HUNT2 (1995–1997) to HUNT3 (2006–2008). However, different methodology was used in defining use of drugs; i.e. self-reported use of anxiolytic and sleep medication in HUNT2 versus prescribed addictive psychotropic drugs among participants in HUNT3. Thus, due to the limitations pointed out we must be cautious in interpreting these results. The combined use of alcohol and drugs with addiction potential among older adults may be the result of using alcohol to self-medicate mental disorders and pain with alcohol, while taking drugs with addiction potential [29]. Nevertheless, we did not find an association between mental health (e.g. anxiety and depression) and the possible combination of frequent drinking and being prescribed drugs with addiction potential. Another explanation might be that older adults are unaware the potential harm of concomitant use [17, 62].

We also found that more men than women in HUNT3 were frequent drinkers, and more women than men were prescribed drugs with addiction potential. These findings are consistent with previous studies [7, 18, 19, 38]. Higher alcohol consumption among men than women in our study may be explained by social norms, cultural values, gender-specific roles in Norway and biological differences between men and women [4, 45]. A previous study showed that among participants 60 years and older in HUNT3, the prevalence of insomnia, musculoskeletal pain, osteoporosis, fibromyalgia and arthrosis was higher in women than men [50], and may explain why women were more likely to be prescribed drugs with addiction potential in our study. Another reason might be that women are much more likely than men to talk about their feelings and distress and to seek help for their health issues [63]. Physicians may also respond differently to mental health and pain problems among women and men [7, 64, 65] and therefore be more likely to prescribe drugs with addiction potential to women [7, 66]. Men might use alcohol to resolve problems with anxiety, depression, insomnia and pain, while women to a greater degree are prescribed drugs with addiction potential to treat these kinds of health problems [63, 66].

In line with previous studies, we found that the prevalence of frequent drinking (≥ 4 days/week) decreased with increasing age [19, 31], while the prevalence of being prescribed drugs with addiction potential increased with increasing age [18, 19, 30, 35, 36, 40]. The reasons why older adults decrease their alcohol consumption as they become older might be because of poorer health, decreased socialization, lower tolerance to alcohol and negative experiences with alcohol [3, 15, 67], a birth cohort effect [3] where older cohorts are likely to drink less than the younger cohort (i.e. the baby boomers born after World War II) [15] and the mortality hypothesis, where those who drink more die at an earlier age due to health problems [15, 20, 21, 27]. The prevalence of insomnia [68], comorbidities [42] and chronic pain [69] increases in older adults and may explain the higher odds of being prescribed drugs with addiction potential by increasing age. Another explanation might be that older adults’ existential needs and mental health problems may be handled by prescribing addictive psychotropic drugs instead of alternative treatment options such as referral to psychotherapy [70, 71]. General practitioners have also reported that it can be difficult to stop the prescription of addictive psychotropic drugs [71] and are likely to renew prescriptions without performing a drug review [72].

Consistent with other studies, more years of education [17, 18, 21, 22, 45, 46] and living in urban areas [73] were associated with frequent drinking in our study. More education and living in urban areas indicate higher socioeconomic status [45], and these older adults may better afford to buy alcohol. Social and cultural norms on drinking patterns, and alcohol availability, i.e. alcohol outlets, pubs and other places that serve alcoholic beverages, may also vary between urban and rural areas [45, 73].

Smokers had higher odds of consuming alcohol frequently, being prescribed drugs with addiction potential, and the possible combination of frequent drinking and use of prescribed drugs with addiction potential than non-smokers. This association has been observed in several previous studies, both for alcohol [9, 17–21, 23] and for addictive psychotropic drugs [35, 39]. Few studies have investigated factors associated with the use of both alcohol and drugs with addiction potential [18, 19, 38], and none of them found an association between smoking and the possible combination of use of alcohol and drugs. Biological factors [74], genetic factors [74] and increased satisfaction and calming effects with combined use [75] may explain the co-use of alcohol and tobacco. Smokers have reported that nicotine reduces their symptoms of anxiety [76] and chronic pain [77] and might be reasons for the association between smoking and drugs with addiction potential in our study.

### Strengths and limitations

This study has several strengths. Firstly, we have followed a large population cohort of community-dwelling adults in Nord-Trøndelag County in Norway [47]. Nord-Trøndelag County has a stable and homogenous population [4, 50], and the county is similar to the general population of Norway in many health aspects [48–50].

A second and major strength of our study is its longitudinal design, spanning more than a decade. This allowed us to investigate several factors potentially associated with the later use of alcohol and/or being prescribed drugs with addiction potential.

Thirdly, we used data from a nationwide prescription database [52] which gave us complete information on all prescriptions dispensed in Norwegian pharmacies during the observation period. This prevents selection bias and recall bias [78]. However, we do not know if the dispensed drugs were taken or the indication for use [78].

Despite the strengths of our study, it still has limitations that should be considered. Firstly, participants who responded to the alcohol frequency question in HUNT2, but not in HUNT3, were excluded from our study. Those excluded, as previously stated, may have quit drinking, and therefore the increased prevalence of frequent drinking in HUNT3 could be overestimated. Even so, a non-participation study conducted after HUNT3 [50] indicate on the other hand that those with the most serious addiction problems did not participate in HUNT3. Thus, it is likely that the prevalence of high drinking frequency and prescription of drugs with addiction potential in HUNT3 were underestimated.

As in other studies, self-report of alcohol consumption may contribute to less accurate estimates and an underestimation of alcohol consumption, and especially in heavy drinkers [20]. Further, the findings in our study represent patterns of alcohol use that are solely based on drinking frequency. We did not have information about how many drinks or units of alcohol the participants consumed the day they drank alcohol. It is therefore unclear if someone who drinks 4 days/week consumes more alcohol than someone who drinks 2 days/week. Moreover, since our definition of frequent drinking only included drinking alcohol 4 days or more a week, this definition may include participants with both risky drinking (≥ 7 drinks/week) and non-risky drinking (< 7 drinks/week) [60].

Variations in how questions about alcohol consumption were asked in HUNT2 and HUNT3 make it necessary to be cautious when interpreting results. In addition, it was not possible to determine whether alcohol and drugs with addiction potential were used simultaneously. Participants may not drink on the day they take the drug, and not all drugs are taken daily. An interaction can only occur if both alcohol and drugs are present in the body simultaneously [79].

Moreover, HUNT2 was conducted in 1995–1997 and HUNT3 in 2006–2008. Thus, a more current survey might have produced different data. The fourth health study in Nord-Trøndelag (HUNT4) (2017–2019) will give us new opportunities to investigate the trend in use of alcohol among older adults in Norway. In the present study we chose to focus on frequent alcohol consumption defined as drinking 4 days or more a week. It could also be of interest to study the trend of other frequency of alcohol consumption.

In addition, very few person 80 years or older in HUNT2 participated in HUNT3. These participants were kept in our analyses as they did not differ compared to those 79 years or younger regarding frequent drinking or the possible combination of frequent drinking and use of drugs with addiction potential.

Although we adjusted for various socio-demographic, physical and psychological health factors, the possibility of residual confounding cannot be ruled out [35]. In our logistic regression model, the R-squared (R^2^) for the outcome possible combination of frequent drinking and being prescribed drugs with addiction potential was very low (6.8%). This indicates that other factors not included in our analyses have great importance for the association with the possible combination of use of alcohol and drugs with addiction potential. We did not have information about insomnia, pain, polypharmacy and loss of close family members and friends, all of which may be potential factors associated with higher use of alcohol and drugs with addiction potential in older adults [18, 19, 28, 35, 36, 39, 40].

### Clinical implications

Knowledge about factors associated with frequent alcohol consumption and drugs with addiction potential in older adults may help health personnel to identify subgroups at higher future risk [15]*.* To avoid potential interaction between alcohol and drugs with addiction potential, there is a need for health personnel to focus on asking older adults about their drinking behaviour and drug use [31]*.* However, prior evidence has shown that health personnel often do not have established routines for asking older patients about their alcohol consumption, or informing them about the health consequences of alcohol consumption at older age and the possible harmful interaction effect between alcohol and drugs [67, 70, 71].

## Conclusion

Individuals who were identified as frequent drinkers in HUNT2 were more likely to be frequent drinkers in HUNT3, and to have the possible combination of frequent drinking and being prescribed drugs with addiction potential in HUNT3.

Understanding factors associated with later use of frequent drinking and the possible combination of frequent drinking and use of prescribed drugs with addiction potential might contribute to increased awareness among health care personnel regarding older patients that could be at higher risk in the future. Health care professionals need to be aware of use of alcohol among older adults using drugs with addiction potential.

## Additional files

Additional file 1: **Table S1.** Sample characteristic of participants in HUNT2 (1995–97) who responded versus did not respond to the alcohol frequency question in HUNT3 (2006–08) (DOCX 16 kb)

Additional file 2: **Table S2.** Unadjusted logistic regression analyses of alcohol consumption and use of prescribed drugs with addiction potential (HUNT3 2006–08). The table shows unadjusted odds ratio for the association between independent variables assessed in HUNT2 (1995–97) (sociodemographic variables, physical and mental health) and three dependent variables assessed in HUNT3 (2006–08): Outcomes 1) Frequent drinking (alcohol consumption ≥ 4 days/week), 2) Prescribed drugs with addiction potential and 3) The possible combination of frequent drinking and being prescribed drugs with addiction potential. (DOCX 16 kb)

## Abbreviations

ATC: Anatomical therapeutic classification system
BZD: Benzodiazepines
CI: Confidence interval
HADS: Hospital Anxiety and Depression Scale
HUNT: The Nord-Trøndelag Health Study
HUNT2: The Nord-Trøndelag Health Study in 1995–1997
HUNT3: The Nord-Trøndelag Health Study in 2006–2008
NorPD: Norwegian Prescription Database
OR: Odds ratio
