# Supplementary material for: Factors associated with alcohol consumption and prescribed drugs with addiction potential among older women and men – the Nord-Trøndelag health study (HUNT2 and HUNT3), Norway, a population-based longitudinal study
Source: BMC Geriatr. 2019 Apr 18;19:113. doi: 10.1186/s12877-019-1114-2 (PMC6472008; doi:10.1186/s12877-019-1114-2)
Supplement: Supplementary file 1 — Table S1. Sample characteristic of participants in HUNT2 (1995–97) who responded versus did not respond to the alcohol frequency question in HUNT3 (2006–08) (DOCX 16 kb) [file 12877_2019_1114_MOESM1_ESM.docx]

**Table S1: Sample characteristic of participants in HUNT2 (1995-97) who responded versus did not respond to the alcohol frequency question in HUNT3 (2006-08)**

Responder to the alcohol Non-responder to the alcohol

HUNT2 question in HUNT3 question in HUNT3 P-value

Overall N (%) 7955 (95.2) 400 (4.8)

Gender

Female N (%) 3876 (48.7) 293 (73.3) < 0.001^a^

Male N (%) 4079 (51.3) 107 (26.7)

Age

53-59 years N (%) 3462 (43.5) 95 (23.8) < 0.001^a^

60-64 years N (%) 2020 (25.4) 88 (22.0)

65 + years N (%) 2473 (31.1) 217 (54.2)

Education

Up to ten year education N (%) 6287 (82.0) 326 (92.3) < 0.001^a^

Vocational and general education N (%) 212 (2.8) 7 (2.0)

College and university N (%) 1168 (15.2) 20 (5.7)

Living in

Rural areas N (%) 3036 (38.2) 181 (45.3) 0.001^a^

Urban areas N (%) 4919 (61.8) 219 (54.7)

Marital status

No living spouse or partner N (%) 1539 (19.4) 118 (29.6) < 0.001^a^

Living spouse or partner N (%) 6405 (80.6) 281 (70.4)

Smoking status

Never smoked daily N (%) 2970 (37.6) 180 (46.3) 0.003^a^

Former daily smoker N (%) 3104 (39.3) 131 (33.6)

Daily smoker N (%) 1820 (23.1) 78 (20.1)

Overall health status

Poor/not quite good N (%) 2574 (32.6) 156 (39.1) 0.007^a^

Good/very good N (%) 5320 (67.4) 243 (60.9)

Hospitalized during the last 5 years N (%) 2262 (32.7) 122 (37.2) 0.093^a^

HADS anxiety score ≥ 8 N (%) 807 (12.2) 44 (17.7) 0.011^a^

HADS depression score ≥ 8 N (%) 861 (11.7) 45 (13.8) 0.237^a^

Life satisfaction

Dissatisfied/neither N (%) 1183 (15.0) 56 (14.3) 0.703^a^

satisfied nor dissatisfied

Satisfied N (%) 6689 (85.0) 335 (85.7)

Alcohol consumption ≥ 4 days/week^1^ N (%) 83 (1.0) 1 (0.3) 0.190^b^

Anxiolytic or sleep medication every week^2^ N (%) 594 (9.0) 45 (15.5) < 0.001^a^

HADS = Hospital Anxiety Depression scale.

^1^Self-reported alcohol consumption assessed among participants in HUNT2.

^2^Information about prescribed drugs in HUNT2: Self-reported use of anxiolytic or sleep medication every week or more in the last month.

^a^Significance testing with Chi Square test

^b^Significance testing with Fisher Exact test
