# Supplementary material for: Factors associated with alcohol consumption and prescribed drugs with addiction potential among older women and men – the Nord-Trøndelag health study (HUNT2 and HUNT3), Norway, a population-based longitudinal study
Source: BMC Geriatr. 2019 Apr 18;19:113. doi: 10.1186/s12877-019-1114-2 (PMC6472008; doi:10.1186/s12877-019-1114-2)
Supplement: Supplementary file 2 — Table S2. Unadjusted logistic regression analyses of alcohol consumption and use of prescribed drugs with addiction potential (HUNT3 2006–08). The table shows unadjusted odds ratio for the association between independent variables assessed in HUNT2 (1995–97) (sociodemographic variables, physical and mental health) and three dependent variables assessed in HUNT3 (2006–08): Outcomes 1) Frequent drinking (alcohol consumption ≥ 4 days/week), 2) Prescribed drugs with addiction potential and 3) The possible combination of frequent drinking and being prescribed drugs with addiction potential. (DOCX 16 kb) [file 12877_2019_1114_MOESM2_ESM.docx]

**Table S2: Unadjusted logistic regression analyses of alcohol consumption and use of drugs with addiction potential (HUNT3 2006-08)**

Outcome 1 (HUNT3): Outcome 2 (HUNT3): Outcome 3 (HUNT3):

Frequent drinking: Prescribed drugs with Possible combination of frequent drinking

alcohol consumption ≥ 4 days/week^1^ addiction potential^2^ (alcohol consumption ≥ 4 days/week) and being prescribed drugs with addiction potential

Unadjusted^a^  Unadjusted^a^  Unadjusted^a^

OR 95% CI OR 95% CI OR 95% CI

HUNT2

Gender (men) **2.14 (1.70-2.70) 0.42 (0.39-0.46)** 1.26 (0.86-1.84)

Age

54-59 1 (ref. category) 1 (ref. category) 1 (ref. category)

60-64 0.80 (0.61-1.05) **1.34 (1.20-1.49)**  0.70 (0.42-1.18)

≥ 65 **0.62 (0.48-0.81)**  **2.06 (1.88-2.27)**  0.93 (0.61-1.43)

Level of education

Up to ten years education 1 (ref. category) 1 (ref. category) 1 (ref. category)

Vocational and general **4.93 (3.13-7.77)** 0.81 (0.61-1.06) **3.30 (1.41-7.71)**

education

College and university **4.80 (3.79-6.09)** **0.69 (0.61-0.78)**  **3.48 (2.30-5.27)**

Living in

Rural areas 1 (ref. category) 1 (ref. category) 1 (ref. category)

Urban areas **2.00 (1.55-2.59)** **0.87 (0.80-0.95)**  1.57 (1.03-2.38)

Marital status

No living spouse or

partner 1 (ref. category) 1 (ref. category) 1 (ref. category)

Living spouse or

partner **1.36 (1.01-1.83)**  **0.76 (0.69-0.84)** 0.82 (0.53-1.28)

Smoking status

Never smoked daily 1 (ref. category) 1 (ref. category) 1 (ref. category)

Former daily smoker **2.51 (1.91-3.29)**  0.97 (0.88-1.06) **2.37 (1.47-3.81)**

Daily smoker **2.10 (1.54-2.88)**  **1.38 (1.24-1.54) 2.46 (1.45-4.17)**

Overall health status

Bad/not quite good 1(ref. category) 1 (ref. category) 1 (ref. category)

Good/very good **1.49 (1.16-1.92) 0.47 (0.43-0.51)** 0.78 (0.53-1.14)

Hospitalized during the

last 5 years 0.96 (0.75-1.24) **1.42 (1.29-1.55)** 1.25 (0.83-1.90)

HADS anxiety score

< 8 1 (ref. category) 1 (ref. category) 1 (ref. category)

≥ 8 0.81 (0.55-1.19) **2.54 (2.23-2.89)** 1.44 (0.82-2.52)

HADS depression score

< 8 1 (ref. category) 1 (ref. category) 1 (ref. category)

≥ 8 0.77 (0.52-1.13) **1.67 (1.48-1.90)**  1.12 (0.62-2.01)

Overall life satisfaction

Dissatisfied/neither 1 (ref. category) 1 (ref. category) 1 (ref. category)

satisfied nor dissatisfied

Satisfied 1.27 (0.91-1.79) **0.60 (0.54-0.67)** 1.06 (0.61-1.83)

Alcohol consumption^3^

< 4 days/week 1 (ref. category) 1 (ref. category) 1 (ref. category)

≥ 4 days/week **46.77 (29.60-73.91)** 0.73 (0.45-1.21) **15.86 (8.29-30.35)**

Use of anxiolytic or sleep

medication every week or more^3^

No 1 (ref. category) 1 (ref. category) 1 (ref. category)

Yes 0.75 (0.48-1.18) **8.07 (6.85-9.51) 2.18 (1.28-3.71)**

*Note*: Bold numbers indicate significant associations. OR = odds ratio; CI = confidence interval; HADS = Hospital Anxiety and Depression scale.

^1^Self-reported alcohol consumption assessed among participants in HUNT3 (2006-2008).

^2^Information on prescribed drugs with addiction potential among participants in HUNT3 (2006-2008) was drawn from the Norwegian Prescription Database. Drugs with addiction potential were defined as at least one prescription of benzodiazepines (BZD), z-hypnotics or opioids in two consecutive years (2005/2006, 2006/2007, 2007/2008 or 2008/2009). BZD defined by N03AE, N05BA and N05CD, z-hypnotics defined by N05CF and opioids by N02A.

^3^Self-reported use of alcohol in the last month and self-reported use of anxiolytic or sleep medication every week or more in the last month among participants in HUNT2.

^a^Unadjusted binary logistic regression analysis. Independent variables included separately. Outcome 1: frequent drinking (alcohol consumption ≥ 4 days/week) (alcohol consumption < 4 days/week reference category). Outcome 2: prescribed drugs with addiction potential (BZD, z-hypnotics or opioids) (no prescribed drugs with addiction potential reference category). Outcome 3: possible combination of frequent drinking (alcohol consumption ≥ 4 days/week) and being prescribed drugs with addiction potential (BZD, z-hypnotics or opioids). Reference category: no frequent drinking, no use of drugs with addiction potential or neither frequent drinking - nor being prescribed drugs with addiction potential.
